# RNA Interference against Platelet-Derived Growth Factor Receptor α mRNA Inhibits Fibroblast Transdifferentiation in Skin Lesions of Patients with Systemic Sclerosis

**DOI:** 10.1371/journal.pone.0060414

**Published:** 2013-04-05

**Authors:** Tong Liu, Jian Zhang, Jing Zhang, Xin Mu, Hui Su, Xiaoding Hu, Wenli Liu, Enbing Zhao, Weimin Li

**Affiliations:** 1 Department of Dermatology, First Affiliated Hospital, College of Medicine, Xi’an Jiaotong University, Xìan City, Shaanxi Province, China; 2 Department of Dermatology, Second Affiliated Hospital, College of Medicine, Xi’an Jiaotong University, Xìan City, Shaanxi Province, China; Centro di Riferimento Oncologico, IRCCS National Cancer Institute, Italy

## Abstract

**Objective:**

To down-regulate expression of mRNA for the platelet-derived growth factor receptor (PDGFR)-α, block the signalling pathway of PDGF and its receptor, and study their influence on fibroblast transdifferentiation to myofibroblasts in systemic sclerosis (SSc).

**Methods:**

Fibroblasts from skin lesions of SSc patients and health adult controls were cultured in vitro, and α-smooth muscle actin (α-SMA) expression was determined by immunocytochemistry. Both groups of fibroblasts were stimulated with PDGF-AA, transforming growth factor β_1_ (TGF-β_1_), and costimulated with PDGF-AA and TGF-β_1_, then PDGFR-α and α-SMA mRNA and protein expression were detected with RT-PCR and WB respectively. Three pairs of siRNAs targeting different PDGFR-α mRNA sequences were synthesized for RNAi. SSc and control fibroblasts were transfected with PDGFR-α siRNA; stimulated with PDGF-AA; and assessed for PDGFR-α and α-SMA mRNA and protein expression.

**Results:**

Although the fibroblasts from both groups had similar morphology, the SSc skin lesions had significantly more myofibroblasts than control skin lesions. PDGF-AA stimulation, TGF-β_1_ stimulation, and costimulation significantly up-regulated PDGFR-α and α-SMA mRNA and protein expression in SSc fibroblasts compared to control (*P*<0.05), and costimulation had the strongest effects (*P*<0.05). All three pairs of siRNAs suppressed PDGFR-α mRNA and protein expression (*P*<0.05), but siRNA_1495_ had the highest gene-silencing efficiency (*P*<0.05). PDGFR-α siRNA attenuated the effects of PDGF-AA through up-regulating PDGFR-α and α-SMA mRNA and protein expression and inhibiting fibroblast transdifferentiation to myofibroblasts in SSc (*P*<0.05).

**Conclusions:**

PDGFR-α over-expression in SSc fibroblasts bound PDGF-AA more efficiently and promoted fibroblast transdifferentiation, which was enhanced by TGF-β_1_. PDGFR-α siRNA down-regulated PDGFR-α expression, blocked binding to PDGF-AA, and inhibited fibroblast transdifferentiation to myofibroblasts.

## Introduction

Systemic sclerosis (SSc) is a connective tissue disease of unknown etiology characterized by progressive fibrosis of skin and internal organs, including skin, lung, and heart [1]. Fibroblasts can be activated in a skin wound and transdifferentiated to myofibroblasts, which are characterized by expression of α-smooth muscle actin (α-SMA) [2]. The persistence of myofibroblasts, which are the main cells that synthesize collagens and other extracellular matrix (ECM) components, is believed to be responsible for SSc [2]. Excess ECM components can deposit in affected tissues, leading to fibrosis and ultimately dysfunction or failure of involved tissues and organs [3].

The activation and transdifferentiation of fibroblasts in SSc may be critically influenced by fibrogenic factors, such as transforming growth factor β_1_ (TGF-β_1_) and platelet-derived growth factor (PDGF) [4], [5]. During the pathogenesis of fibrotic diseases, PDGF isoforms secreted by a variety of cell types can stimulate the replication, survival, and migration of myofibroblasts [6]. During tissue fibrosis of liver, lung, kidney and skin, four PDGF isoforms (PDGF-A, B, C, and D) on the surface of myofibroblasts take part in signal transduction through their α or β receptors with intrinsic tyrosine kinase activity [7]. A viable approach for SSc treatment is to suppress the expression of PDGF and PDGFR, thus blocking the signal transduction within cells to reduce transdifferentiation of fibroblasts to myofibroblasts [4]. Recently, there are numerous reports on the application of RNAi to treat fibrotic diseases, such as liver fibrosis, with the effects verified in animal models [8].

The aims of this study were to study the effects of down-regulating PDGFR α mRNA expression and blocking the PDGF-A/PDGFR-α signalling pathway on transdifferentiation of fibroblasts to myofibroblasts in SSc. It was suggested that the PDGFR-α over-expression on the surface of fibroblasts in the skin lesions of SSc bound PDGF-AA ligands more efficiently and promoted fibroblast transdifferentiatiation to myofibroblast, which was enhanced by TGF-β_1_. The PDGFR-α siRNA designed and selected down-regulated the PDGFR-α mRNA expression and blocked the binding to PDGF-AA, and inhibited the process of fibroblast transdifferentiation to myofibroblast.

## Materials and Methods

### Biopsy Specimens

Seven patients with SSc were identified according to the criteria of the American Rheumatism Association (1980), and 7 age- and sex-matched healthy donors were enrolled as controls. Patients were excluded if they had received anti-fibrotic treatment within the previous 6 months. Patients included 3 males and 4 females, with a mean age of 36.29 (range = 29 to 45) and a mean disease duration of 3.86 years (range = 2 to 7). Biopsy specimens were taken from SSc patients’ forearm skin with edema and sclerotic stages, while control specimens were biopsied from healthy adults’ normal forearm skin.

The biopsy specimens were taken after all SSc patients and controls had agreed to participate in the study and had signed their consent forms. Before starting the study, we consulted with our institutional review board, which informed us that approval from the ethics committee was not required but patient agreement was necessary when taking biopsy specimens, according to the related law and rules of our country. [Order of the State Council of the People's Republic of China (No. 149) –– No. 33 of the medical institution regulations; Ministry of Health Order of the People's Republic of China (No. 35) –– No. 61 and 62 of the implementation rules of the medical institution regulations.].

### Main Equipment

CO_2_ cell incubator (Thermo Forma), inverted phase contrast microscope (Nikon), electronic scale (Mettler Toledo), centrifuge (Eppendorf), PCR amplification instrument (Applied Biosystems), and visible spectrum spectrophotometer (Coulter).

### Main Reagents

DMEM medium (Hyclone), fetal bovine serum and Opti-MEM-1 (Gibco), α-SMA monoclonal antibody and TGF-β_1_ (PeproTech), antihuman Vimentin monoclonal antibody (Sigma-Aldrich), S-ABC kit and DAB kit (Boster), Trizol kit (Invitrogen), RT-PCR kit (Fermentas), PDGFR-α siRNA (Shanghai Gene Pharma) and X-treme Gene RNA transfection kit (Roche).

### Cell Culture

Dermal tissue was separated from skin biopsy specimens, and the cells including fibroblasts were suspended in phosphate buffered solution (PBS). Fibroblasts grew in DMEM medium supplemented with 10% fetal bovine serum, and penicillin of 100 U/ml, and streptomycin of 100 mg/ml at 37°C in 5% CO_2_ atmosphere and 95% humidity in 25 cm^2^ culture dishes. For experiments, cells from passages 3 to 7 were used.

### Immunocytochemistry Staining

Sterile coverslips soaked with acid were placed in 6-well culture plates, and both groups of fibroblasts (SSc and control) were seeded in 6-well plates at a density of 1×10^5^/ml. These cells were fixed for 15 minutes with paraformaldehyde and permeabilized with acetone. The diluted monoclonal antibodies of mouse antihuman Vimentin and α-SMA were separately dripped onto fixed cells by the S-ABC method of immunocytochemistry, and finally stained with 3,3'-diaminobenzidine (DAB).

### Fibroblast Stimulation with TGF-β_1_ and PDGF-AA

Fibroblasts were cultured in 6-well plates at a density of 5×10^6^/ml and at 37°C in 5% CO_2_ atmosphere and 95% humidity for 24 hours. When cells reached confluence, the medium was replaced with serum-free medium (SFM) and cultured for 24 hours. For stimulation, both groups of fibroblasts were washed with PBS, and treated by dripping SFM containing 10 ng/ml TGF-β_1_ (T group), 25 ng/ml PDGF-AA (P group), and 10 ng/ml TGF-β_1_ plus 25 ng/ml PDGF-AA (T+P group, namely costimulation). Another group of fibroblasts (untreated group) was cultured with SFM containing free cytokines. These fibroblasts were continuously cultured under the same conditions for 48 hours.

### RNA Interference Experiments

Human PDGFR-α mRNA sequences were obtained from Gene Bank (Gene ID:5156) and three pairs of interfering targets were designed, and no homologous sequences were found with NCBI BLAST. Three pairs of siRNAs (siRNA-I, siRNA-II, siRNA-III) specifically targeting different sequences of PDGFR-α mRNA were synthesized by Shanghai Gene Pharma. The sequences of the three siRNAs were as follows: siRNA-I (sense GUG GCC AUU AUA CUA UUG Utt, antisense ACA AUA GUA UAA UGG CCA Ctt); siRNA-II (sense GGC CAC AUU UGA ACA UUG Utt, antisense ACA AUG UUC AAA UGU GGC Ctt); siRNA-III (sense GCU GCC UGG ACA AUA UAA Att, antisense UUU AUA UUG UCC AGG CAG Ctt); negative control siRNA (sense UUC UCC GAA CGU GUC ACG Utt, antisense ACG UGA CAC GUU CGG AGA Att). Fibroblasts from skin lesions of SSc patients were cultured in 6-well plates at a density of 2×10^5^/ml, 2 ml each well, at 37°C in 5% CO_2_ atmosphere and 95% humidity in incubator. When the cells reached 50% to 70% confluence, they were transfected with siRNA using X-treme GENE siRNA Transfection kit following the manufacturer’s recommendations and the culture continued. At 48 h post-transfection, the cells were checked for both PDGFR-α mRNA and α-SMA mRNA expression with reverse transcription-polymerase chain reaction (RT-PCR) assay.

### RT-PCR Experiments

Total RNA was extracted from cultured fibroblasts with Trizol kit after stimulating with TGF-β_1_ alone, PDGF-AA alone, costimulating with both TGF-β_1_ and PDGF-AA, and transfecting with siRNA. RT-PCR test was performed with an RT-PCR kit. Briefly, 2 µg total RNA from each sample was reversely transcribed to cDNA with Oligo (dT) as primers. The RT reaction was done at 42°C for 50 minutes in a volume of 20 µL, and 2 µL of each RT solution was then used for PCR in a volume of 50 µL. α-SMA mRNA primers (sense: 5′ CTG GAC GCA CAA CTG GCA TC 3′; antisense: 5′ TCA GCA GTA GTA ACG GAA TAG C 3′), PDGFR-α mRNA primers (sense: 5′ TGG GAG GGT GGT CTG GAT GAG 3′; antisense: 5′ GAT GAA GGT GGA ACT GCG GGA AC 3′) and β-actin mRNA primers (sense: 5′ TGG GAG GGT GGT CTG GAT GAG 3′; antisense: 5′ GAT GAA GGT GGA ACT GCG GGA AC 3′) were used. The annealing temperature was at 55°C and 30 cycles of amplification were performed. The PCR products were separated by 1.5% agarose gel electrophoresis, collected, and semiquantitatively analyzed by software. Expression intensity was identified by the photodensity ratio of the target gene and β-actin mRNA in the PCR products, namely the relative gray scale (RGS).

### Western Blot Experiments

For Western blot (WB) analysis, cultured fibroblasts were collected by centrifugation, and then were lysed in RIPA lysis buffer after stimulating with TGF-β_1_ alone, PDGF-AA alone, costimulating with both TGF-β_1_ and PDGF-AA, and transfecting with siRNA. Insoluble material was removed by centrifugation at 12,000 rpm for 15 min at 4°C. Subsequently, cell lysates were subjected to electrophoresis using 10% sodium dodecyl sulfate - polyacrylamide (SDS-PA) gels and transferred to a nitrocellulose membrane. Membranes were blocked for 2 hours in 5% nonfat dry milk in TBST (10 mM Tris-HCl and 0.05% Tween 20). The membrane was incubated with primary antibodies overnight at 4°C and then incubated with secondary antibody for 2 hours at room temperature. The primary monoclonal antibodies included mouse monoclonal anti-human α-SMA (1∶1000, PeproTech), mouse monoclonal anti-human PDGFR-α (1∶1000, Sigma-Aldrich) and anti-β-actin (1∶5,000; Sigma-Aldrich). These membranes were incubated in the dark with ECL (Amersham) for chemiluminescence detection. The luminescent signal was detected by CCD camera, recorded and quantified with the Syngene G Box (Syngene, UK). The data were sent to the computer for analysis and documentation.

### Statistical Analysis

The data are presented as means ± standard deviation (SD). Differences in the means of two independent samples with normal distribution were tested for significance by Student’s *t-*test. The means among multiple independent samples with homoscedasticity and normal distribution were compared by ANOVA. When several samples represented continous variables with normal distribution, their relationships were analyzed by Pearson’s correlation coefficient. Differences were considered statistically significant when P<0.05.

## Results

### SSc and Control Skin Fibroblasts have Similar Morphology, but Only SSc Fibroblasts Express α-SMA

The fibroblasts cultured in vitro from skin lesions of SSc patients and normal adult skin were almost morphologically similar and had spindle shapes as well as whirlpool arrangements. Spindle or polygonal cells with larger blue-violet nuclei and more pink cytoplasm were seen with hematoxylin and eosin (HE) staining under light microscopy.

Fibroblasts from SSc patients and controls were inspected for Vimentin with S-ABC immunocytochemistry. Both groups of cells had almost the same morphology, with spindle or polygonal cells showing larger non-staining nuclei and more brown-yellow granular cytoplasm. In both groups of fibroblasts, Vimentin was identified in the cytoplasm of all fibroblasts.

The fibroblasts from skin lesions of SSc patients were shown by S-ABC immunocytochemistry to be positive for α-SMA expression, with granular shape and diffuse distribution in the cytoplasm, while α-SMA expression was absent in the fibroblasts from normal skin.

### PDGFR-α and α-SMA mRNA and Protein Expression is Up-regulated in SSc Fibroblasts Stimulated with TGF-β_1_ and PDGF-AA

**Figure 1:**
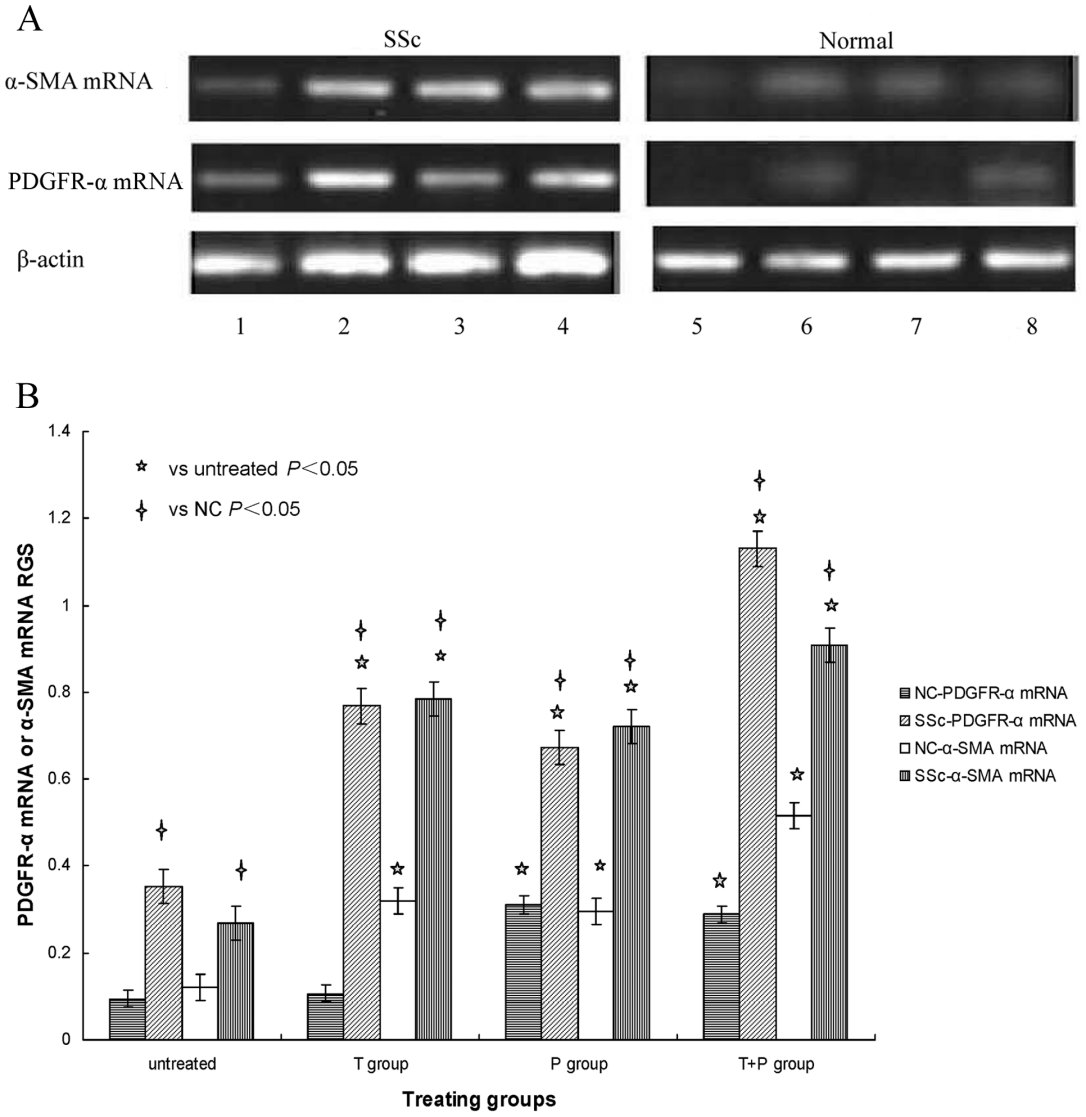
A: PDGFR-α and α-SMA mRNA expression stimulated with TGF-β_1_ and PDGF-AA. SSc and normal skin fibroblast cultures were left untreated (columns 1, 5), costimulated with 10 ng/ml TGF-β_1_ plus 25 ng/ml PDGF-AA (columns 2, 6), stimulated with 10 ng/ml TGF-β_1_ (columns 3, 7) and stimulated with 25 ng/ml PDGF-AA (columns 4, 8). PDGFR-α mRNA and α-SMA mRNA expression was determined by RT-PCR. β-actin mRNA served to normalize expression of mRNA. B: Quantification of the data. The relative gray scale (RGS) for PDGFR-α mRNA and α-SMA mRNA expression by cultured fibroblasts from SSc skin lesions and normal skin left untreated or stimulated with 10 ng/ml TGF-β_1_ (T group), 25 ng/ml PDGF-AA (P group), or 10 ng/ml TGF-β_1_ plus 25 ng/ml PDGF-AA (T+P group). PDGFR-α mRNA and α-SMA mRNA expression is up-regulated in SSc fibroblasts stimulated with TGF-β_1_ and PDGF-AA.

**Figure 2:**
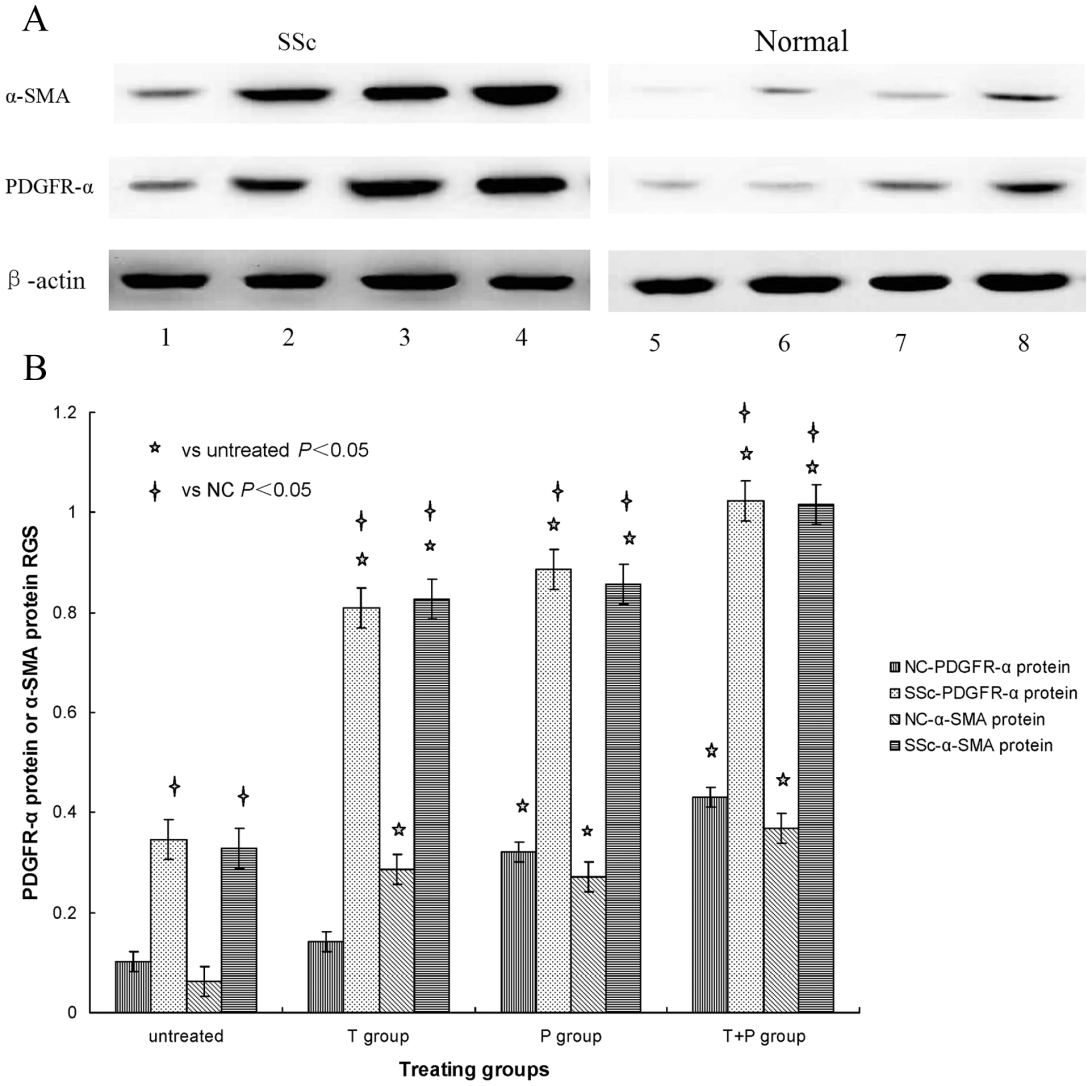
A: PDGFR-α and α-SMA protein expression stimulated with TGF-β_1_ and PDGF-AA. SSc and normal skin fibroblast cultures were left untreated (columns 1, 5), costimulated with 10 ng/ml TGF-β_1_ plus 25 ng/ml PDGF-AA (columns 2, 6), stimulated with 10 ng/ml TGF-β_1_ (columns 3, 7) and stimulated with 25 ng/ml PDGF-AA (columns 4, 8). PDGFR-α protein and α-SMA protein expression was determined by WB. β-actin served to normalize expression of protein. B: Quantification of the data. RGS for PDGFR-α protein and α-SMA protein expression by cultured fibroblasts from SSc skin lesions and normal skin left untreated or stimulated with 10 ng/ml TGF-β_1_ (T group), 25 ng/ml PDGF-AA (P group), or 10 ng/ml TGF-β_1_ plus 25 ng/ml PDGF-AA (T+P group). PDGFR-α protein and α-SMA protein expression is up-regulated in SSc fibroblasts stimulated with TGF-β_1_ and PDGF-AA.

SSc and control fibroblasts were stimulated with 10 ng/ml TGF-β_1_ (T group), 25 ng/ml PDGF-AA (P group), and 10 ng/ml TGF-β_1_ plus 25 ng/ml PDGF-AA (T+P group), and PDGFR-α and α-SMA mRNA and protein expression was measured by RT-PCR and WB respectively. Figure 1A and figure 2A shows specific bands for both PDGFR-α and α-SMA mRNA and protein expression in all treatment groups separately, with more obvious expression in SSc fibroblasts. Figure 1B and figure 2B shows the relative gray scale (target mRNA and protein expression normalized to that of β-actin) to quantify the results shown in figure 1A and figure 2A respectively.

In SSc fibroblasts, both PDGFR-α and α-SMA mRNA and protein expression in all three treatment groups (T, P, and T+P) were significantly higher than in the untreated group (*P*<0.05). These results demonstrate that stimulation with PDGF-AA and TGF-β_1_ alone as well as costimulation with both growth factors increased PDGFR-α and α-SMA mRNA and protein expression in SSc fibroblasts, and costimulation had the strongest effects.

In control fibroblasts, comparison with the untreated group shows that PDGFR-α mRNA and protein expression was significantly higher in the P and T+P groups (*P*<0.05), was not significantly affected in the T group (*P*>0.05), and α-SMA mRNA and protein expression was significantly higher in all three groups (*P*<0.05). These results show that TGF-β_1_ had no effect on PDGFR-α mRNA and protein expression but increased α-SMA mRNA and protein expression in normal skin fibroblasts, while PDGF-AA stimulation and PDGF-AA+TGF-β_1_ costimulation up-regulated PDGFR-α and α-SMA mRNA and protein expression, with the strongest effects after costimulation.

The results also show that both PDGFR-α and α-SMA mRNA and protein expression in SSc fibroblasts was significantly higher than in control fibroblasts after the same stimulation conditions or when not treated (*P*<0.05). These results suggest that PDGF-AA up-regulated PDGFR-α over-expression on the surface of SSc fibroblasts to initiate signal transduction via PDGF-A/PDGFR-α and to promote fibroblast trandifferentiation to myofibroblasts in skin lesions of SSc patients, and these effects were enhanced by TGF-β_1_.

The expression of both PDGFR-α and α-SMA mRNA and protein in SSc fibroblasts increased gradually after no treatment or stimulation with PDGF-AA, TGF-β_1,_ and PDGF-AA plus TGF-β_1_. Analysis of the relationship between PDGFR-α and α-SMA mRNA and protein expression showed that it was highly significantly and positively correlated (r = 0.925, *P*<0.05; r = 0.863, *P*<0.05) separately. These results suggest that PDGF-AA increased significantly the binding to PDGFR-α and promoted fibroblast transdifferentiation to myofibroblasts in SSc skin lesions.

### PDGFR-α siRNA Blocks Fibroblast Transdifferentiation in SSc

To determine the effect of blocking PDGFR-α expression on fibroblast transdifferentiation to myofibroblasts in SSc, the same numbers of SSc fibroblasts were transfected for 48 hours with siRNA-I (siRNA_1495_), siRNA-II (siRNA_2284_), siRNA-III (siRNA_3188_), siRNA-NC (normal control), and normal control. These five groups of fibroblasts were examined for RNA intactness by degeneration gel electrophoresis of total RNA. All five groups showed two clear bands at 28 S and 18 S, with no obvious diffused traces in the gel, and a luminance ratio for the 28 S and 18 S bands of about 2 to 1 (data not shown). These results demonstrated that total RNA was completely extracted.

We then determined the inhibition rate for PDGFR-α mRNA and protein expression by the following formula:

Inhibition rate = [normal control (X)-siRNA (X)]/normal control (X).

**Figure 3:**
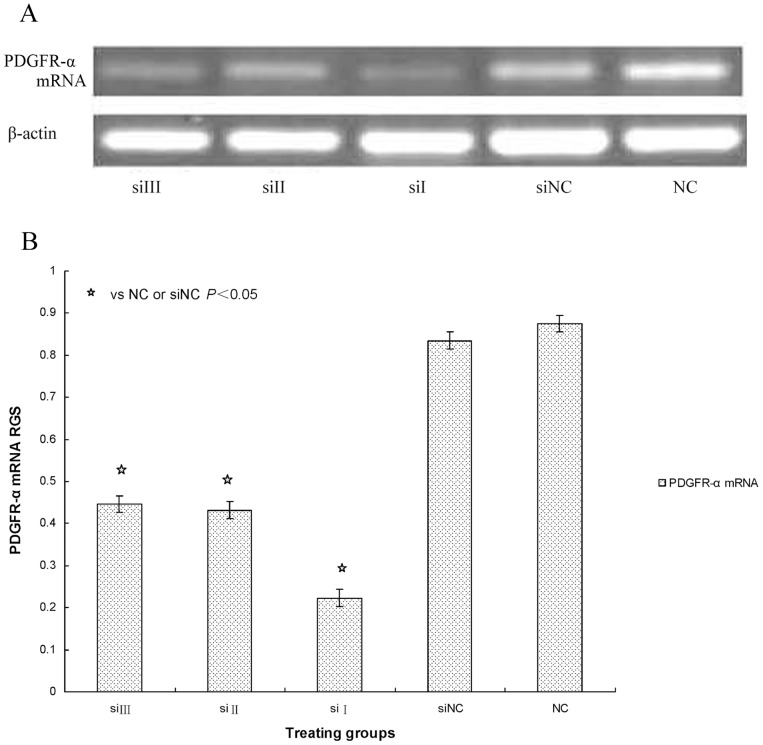
A: PDGFR-α mRNA expression transfected with siRNA. The PDGFR-α mRNA expression was seen after transfection with PDGFR-α siRNA. SSc fibroblast cultures were left siRNA III or siRNA_3188_ (siIII), siRNA II or siRNA_2284_ (siII), siRNA I or siRNA_1495_ (siI), siRNA negative control (siNC), and untreated fibroblasts (NC). PDGFR-α mRNA expression was determined by RT-PCR. β-actin mRNA served to normalize expression of mRNA. B: Quantification of the data. RGS for PDGFR-α mRNA expression by cultured fibroblasts from SSc skin lesions left siRNA III or siRNA_3188_ (siIII), siRNA II or siRNA_2284_ (siII), siRNA I or siRNA_1495_ (siI), siRNA negative control (siNC), and untreated fibroblasts (NC). PDGFR-α mRNA expression was significantly suppressed in fibroblasts transfected with siRNA.

**Figure 4:**
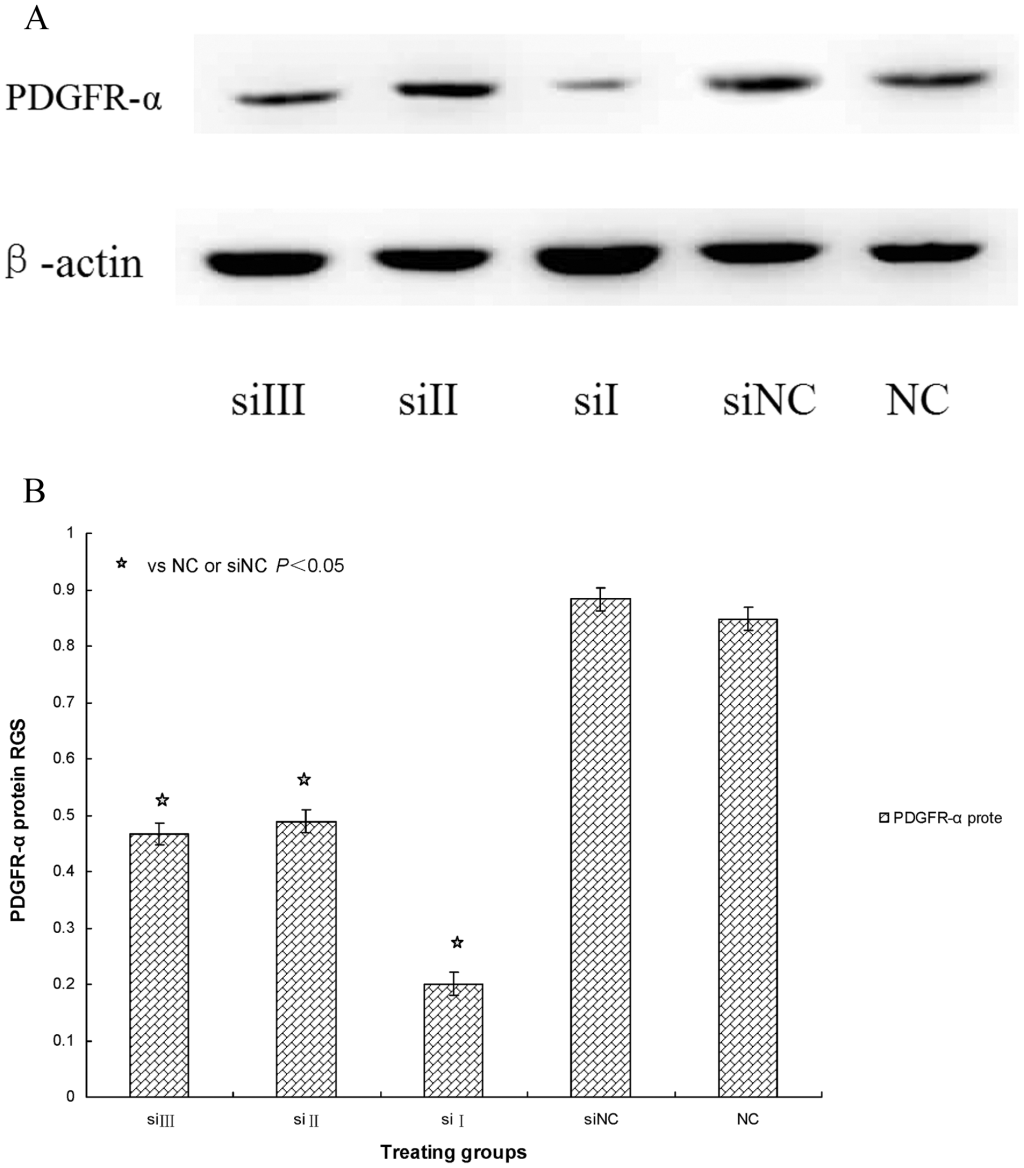
A: PDGFR-α protein expression transfected with siRNA. The PDGFR-α protein expression was seen after transfection with PDGFR-α siRNA. SSc fibroblast cultures were left siRNA III or siRNA_3188_ (siIII), siRNA II or siRNA_2284_ (siII), siRNA I or siRNA_1495_ (siI), siRNA negative control (siNC), and untreated fibroblasts (NC). PDGFR-α protein expression was determined by WB. β-actin served to normalize expression of protein. B: Quantification of the data. RGS for PDGFR-α protein expression by cultured fibroblasts from SSc skin lesions left siRNA III or siRNA_3188_ (siIII), siRNA II or siRNA_2284_ (siII), siRNA I or siRNA_1495_ (siI), siRNA negative control (siNC), and untreated fibroblasts (NC). PDGFR-α protein expression was significantly suppressed in fibroblasts transfected with siRNA.

The inhibition rates after culturing cells for 48 hours with siRNA-I, siRNA-II and siRNA-III were 75%, 54%, and 52%, respectively. These data show that the three pairs of PDGFR-α siRNAs inhibited PDGFR-α mRNA and protein expression to different degrees when compared with expression in the siRNA-NC and normal control groups (*P*<0.05) respectively. These results indicate that PDGFR-α siRNA-I (siRNA_1495_), which was designed and synthesized against the 1495 sequence of PDGFR-α mRNA, had the strongest suppression effects and the highest gene-silencing efficiency in the three siRNAs (*P*<0.05) (Figure 3A, 3B, 4A and 4B). Therefore, siRNA-I (siRNA_1495_) was selected to transfect cells for subsequent experiments.

**Figure 5:**
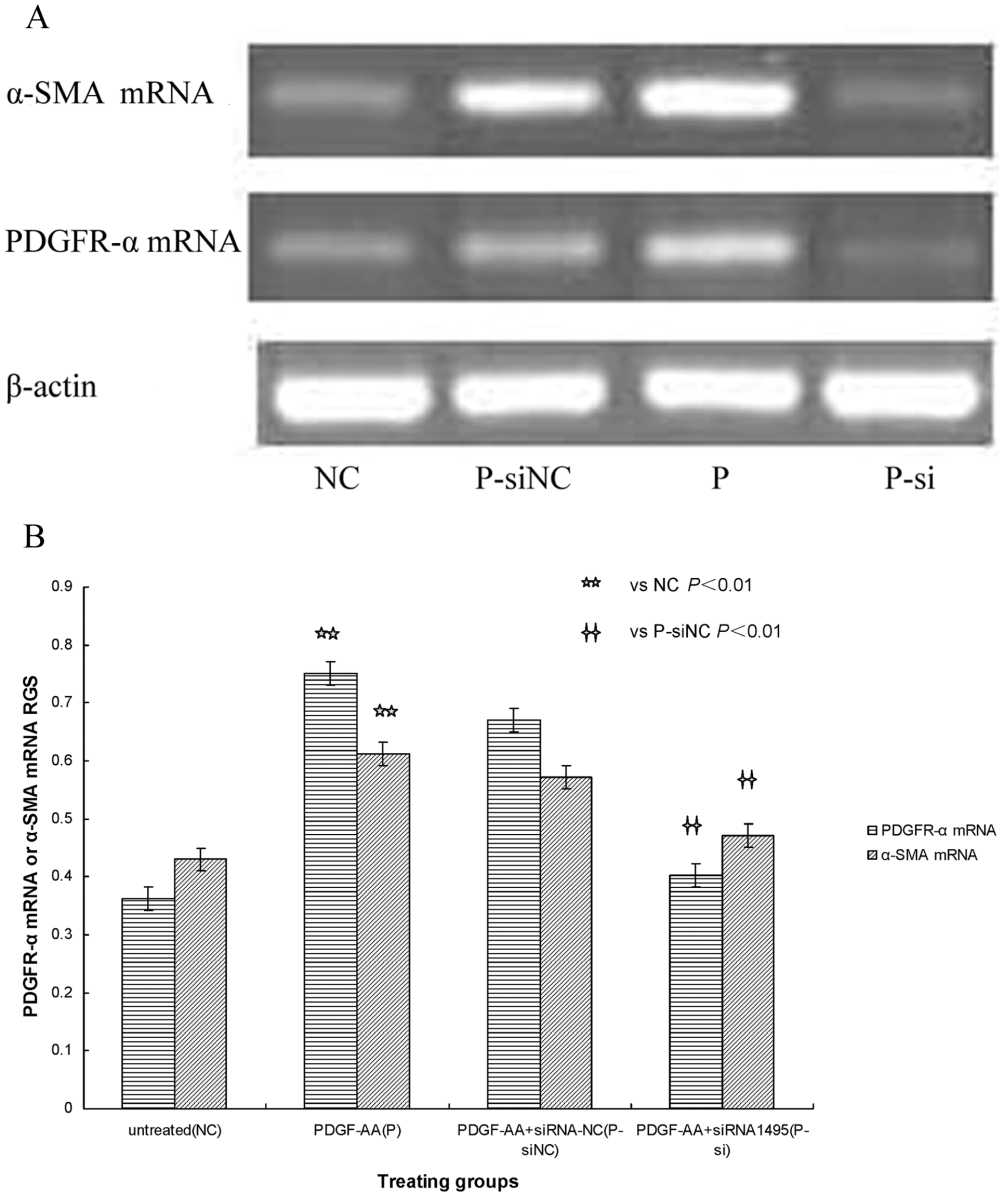
A: PDGFR-α and α-SMA mRNA expression with PDGFR-α siRNA. RGS for PDGFR-α mRNA and α-SMA mRNA expression by cultured fibroblasts from SSc skin lesions left untreated fibroblasts (NC), fibroblasts treated with PDGF-AA+siRNA negative control (P-siNC), fibroblasts stimulated with PDGF-AA (P), and fibroblasts treated with PDGF-AA+PDGFR-α siRNA (P-si). PDGFR-α mRNA and α-SMA mRNA expression was determined by RT-PCR. β-actin mRNA served to normalize expression of mRNA. B: Quantification of the data. PDGFR-α siRNA reduces PDGFR-α mRNA and α-SMA mRNA expression in SSc fibroblasts.

**Figure 6:**
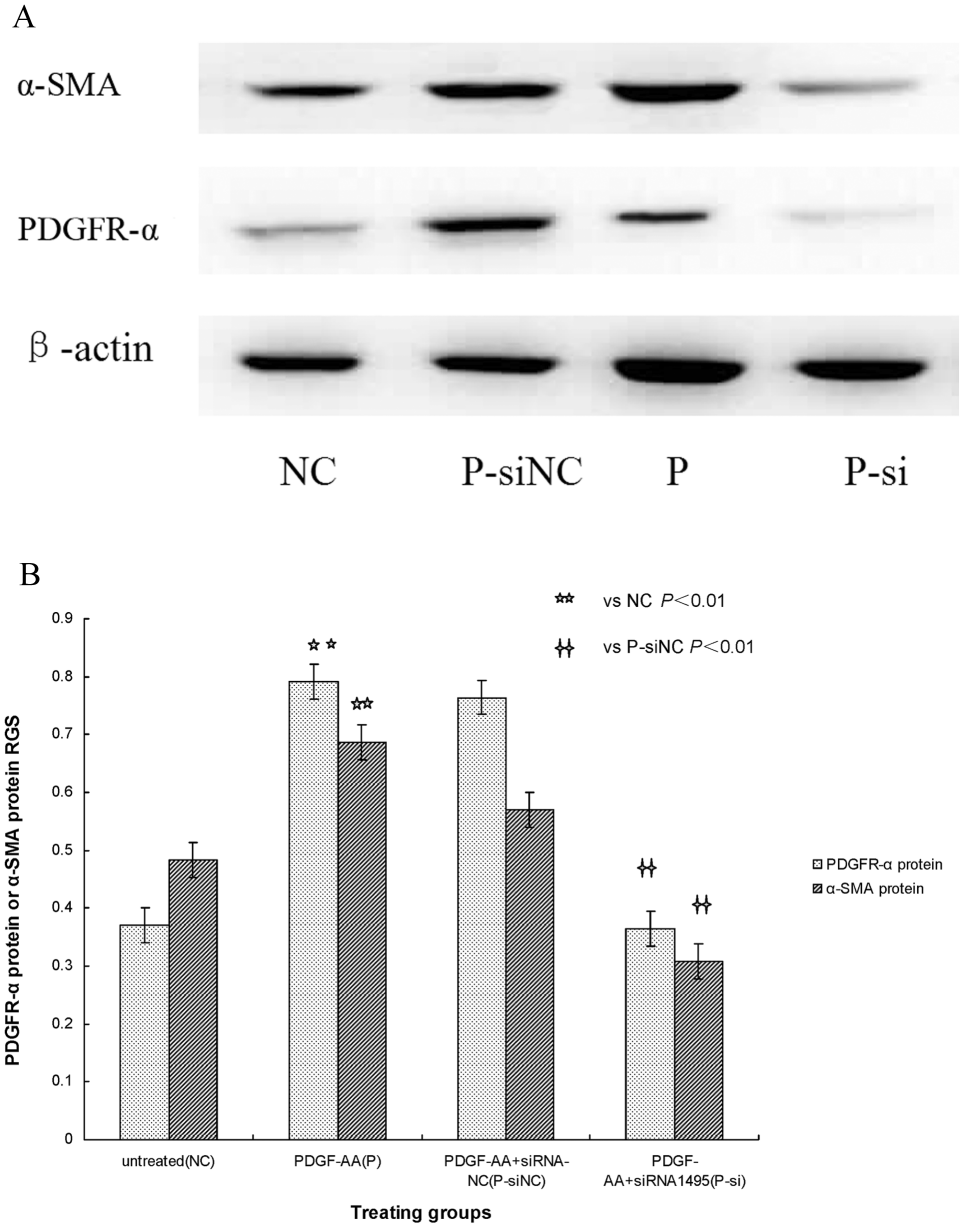
A: PDGFR-α and α-SMA protein expression with PDGFR-α siRNA. RGS for PDGFR-α protein and α-SMA protein expression by cultured fibroblasts from SSc skin lesions left untreated fibroblasts (NC), fibroblasts treated with PDGF-AA+siRNA negative control (P-siNC), fibroblasts stimulated with PDGF-AA (P), and fibroblasts treated with PDGF-AA+PDGFR-α siRNA (P-si). PDGFR-α protein and α-SMA protein expression was determined by WB. β-actin served to normalize expression of protein. B: Quantification of the data. PDGFR-α siRNA reduces PDGFR-α protein and α-SMA protein expression in SSc fibroblasts.

Indeed, we found that compared with the siRNA-NC transfection group, both PDGFR-α and α-SMA mRNA and protein expression, which increased with PDGF-AA treatment, were significantly attenuated when transfected with PDGFR-α siRNA_1495_ (*P*<0.01) (Figure 5A, 5B, 6A and 6B). Analysis of the relationship between PDGFR-α and α-SMA mRNA and protein expression showed that expression of these mRNAs and proteins was highly significantly and positively correlated (r = 0.916, *P*<0.05; r = 0.877, *P*<0.05) separately. These results suggest that PDGFR-α mRNA and protein expression was blocked by PDGFR-α siRNA, and α-SMA mRNA and protein expression was down-regulated. These results also show that the effects of PDGF-AA, which significantly promoted fibroblast transdifferentiation to myofibroblasts in skin lesions of SSc patient-derived cultures, were obviously inhibited by PDGFR-α siRNA.

## Discussion

The important patho-physiological features of SSc are vasculopathy, immune system disorders, and abnormal ECM deposition [3]. Early manifestations of the disease are dominated by vasculopathy with capillary rarefaction and inflammatory autoimmune processes, while the key feature of late-stage disease is the onset of fibrosis, which can cause organ failure and accounts for much of the morbidity and mortality in SSc patients [3]. Although antifibrotic therapies that selectively target fibroblast activation are not yet available, reducing fibroblast transdifferentiation can contribute to blocking ECM production and may become a viable approach for SSc treatment [4].

ECM components are well known to be produced by myofibroblasts, which are characterized by expression of α-SMA [2]. In this study, we showed by immunocytochemistry staining that SSc fibroblasts cultured in vitro could transdifferentiate myofibroblasts expressing α-SMA. Indeed, our results showed similar morphology for both groups of cultured fibroblasts, but α-SMA staining with brown granules were detected in the cytoplasm of fibroblasts from skin lesions of SSc patients, while no α-SMA staining was seen in normal adult skin under the same culture conditions. These results indicated fibroblast transdifferentiation to myofibroblasts in SSc skin-lesion cultures, similar to reported results [9]. The persistence of myofibroblasts, as the ultimate target cells of abnormal immune factors or cytokines, is generally acknowledged to be responsible for scar formation and fibrotic disorders including SSc [2].

Two central mediators in fibrotic diseases, including SSc, are TGF-β_1_ and PDGF [5], [6]. Both growth factors are expressed in the skin lesions of SSc patients and can promote fibroblast activation [9], [10]. In our study, we found that after cultured fibroblasts from skin lesions of SSc patients and normal adult skin were stimulated with TGF β_1_, PDGF-AA, and both factors, PDGFR-α mRNA and protein expression was up-regulated. These results indicate that PDGF-AA binding to PDGFR-α was increased on the surface of SSc fibroblasts to take part in signal transduction via PDGF-A/PDGFR-α and to promote fibroblast trandifferentiation to myofibroblasts at RNA and protein levels, while these effects could be enhanced by TGF-β_1_. Together with previous results [11], our results suggest that fibroblast transdifferentiation to myofibroblasts in SSc operates via an autocrine PDGF-A/PDGFR-α loop. Similarly, PDGF/PDGFR over-expression has been reported in pulmonary fibrosis, glomerulonephritis, cirrhosis and other fibrotic diseases [6]. Therefore, blockade of PDGF signaling has been well documented to reduce the development of fibrosis in various experimental models [12], [13].

Insights have been recently generated into how TGF-β_1_ and PDGF contribute to myofibroblast differentiation [3]. PDGF has been shown to cooperate with TGF-β_1_ in the development of organ fibrosis, and TGF-β_1_ can increase fibroblast sensitivity to PDGF and up-regulate PDGFR-α expression in SSc, thereby creating an autocrine PDGF-A/PDGFR-α loop [5], [6]. Our study results also suggest that in SSc fibroblasts, PDGF-AA, TGF-β_1,_ and costimulation with both factors all increased both PDGFR-α and α-SMA mRNA and protein expression, and costimulation had a synergistic effect. However, in control fibroblasts, both PDGFR-α and α-SMA mRNA and protein expression were up-regulated after stimulation with PDGF-AA and costimulation with both factors, but PDGFR-α mRNA and protein expression was not significantly affected by stimulation with TGF-β_1_ alone. These results suggest that TGF-β_1_ improved cultured fibroblast sensitivity to PDGF-AA and increased PDGFR-α mRNA and protein expression in SSc skin-lesion cultures, but normal fibroblasts were barely affected by TGF-β_1_. These results are similar to those of a previous report [14]. In an early study [15], TGF-β_1_ rendered scleroderma skin fibroblasts more sensitive to PDGF and up-regulated their PDGFR expression. Similar results were later observed by other researchers [16]. TGF-β_1_ stimulates myofibroblasts to synthesize ECM by inducing expression of ECM components such as collagen and fibronectin, by suppressing several matrix metalloprotenases, and by inducing tissue inhibitors of matrix metalloprotenases [5]. Inhibiting TGF-β_1_ and PDGF signaling has been shown to reduce the development of fibrosis in various experimental models [12], [13].

RNAi functions in vivo to block gene expression with double-stranded RNA, which is digested to form many small interfering RNA (siRNA) fragments, which promote mRNA degradation through the introduction of siRNA and combination with homologous sequences in mRNA, thus efficiently and specifically silencing gene expression and eliminating any specific gene phenotype expression [17]. Therefore, RNAi can be used as a simple and effective tool to replace gene knockout and is an effective method for studying the mechanisms of cellular signal transduction [17]. At present, many studies are investigating the possibility of treating SSc patients by blocking the PDGFR with the tyosine kinase inhibitor, imatinib [18], [19].

In our RNAi studies, we first inspected cultured fibroblasts for total RNA intactness after completely extracting RNA. To use the RNAi technique, we selected PDGFR-α mRNA as the target, and three pairs of siRNAs, a negative control, and a normal control were designed and synthesized. Testing these siRNAs for interference effectiveness showed that PDGFR-α mRNA and protein expression was most significantly suppressed in fibroblasts transfected with siRNA-I (siRNA_1495_). Transfecting this siRNA into SSc fibroblasts efficiently suppressed PDGFR-α mRNA and protein expression, thus blocking PDGF-AA binding to PDGFR-α on the surface of activated fibroblasts. We also found that SSc fibloblasts treated in vitro with this siRNA to PDGFR-α mRNA suppressed α-SMA mRNA and protein expression. These results suggest that fibroblast transdifferentiation to myofibroblasts is suppressed by PDGFR-α siRNA. Therefore, blockade of fibroblast transdifferentiation to myofibroblasts with PDGFR-α siRNA might have attenuated ECM deposition and postponed the fibrotic progress in SSc skin-lesion cultures. Similarly, the progress of fibrosis in SSc was attenuated by applying RNAi to inhibit the transcription factor Fra-2 of the ERK signalling pathway between TGF-β_1_ and PDGF [20].

In general, the data presented here provide evidence that PDGFR-α siRNA is an effective gene therapeutic method against fibrosis, reduces PDGFR-α mRNA and protein expression, and inhibits the activation and transdifferentiation of fibroblasts in SSc skin lesions. Because fibroblast transdifferentiation to myofibroblast, which produces main ECM components, takes the important part in the fibrosis in SSc, and affected by the signal pathway of PDGF and PDGFR, the suppression of the pathway through introducing PDGFR-α siRNA for the gene silencing and then blocking PDGFR-α mRNA and protein expression may be to become a viable approach for the treatment. Therefore, the beneficial effects of this RNAi technique will open novel avenues for research on SSc treatments.
